# Structural and functional foot disorders in patients with genodermatoses: a single-centre, retrospective chart review

**DOI:** 10.1186/s13023-022-02207-x

**Published:** 2022-02-16

**Authors:** Aldona Pietrzak, Bartlomiej Wawrzycki, Matthias Schmuth, Katarzyna Wertheim-Tysarowska

**Affiliations:** 1 Department of Dermatology, Venereology, and Paediatric Dermatology, Medical University of Lublin, Staszica 11, 20-080 Lublin, Poland; 2 Department of Dermatology, Venereology, and Allergy, Medical University Innsbruck, Innsbruck, Austria; 3 Department of Medical Genetics, Institute of Mother and Child, Warsaw, Poland

**Keywords:** Foot disorders, Autosomal recessive congenital ichthyosis, Palmoplantar keratodermas, Ichthyosis follicularis, Atrichia and photophobia, Ectrodactyly-ectodermal dysplasia-clefting, Autosomal dominant ichthyosis with confetti

## Abstract

**Background:**

Skin lesions on the feet and foot deformities impair daily activities and decrease quality of life. Although substantial foot deformities occur in many genodermatoses, few reports have been published on this topic. Therefore, we performed a retrospective chart review to identify patients with genodermatoses and foot disorders. We included 16 patients, who were investigated clinically and with molecular biology.

**Results:**

The following genodermatoses with foot deformities were detected: autosomal recessive congenital ichthyosis (ARCI, n = 7); palmoplantar keratodermas (PPKs, n = 6); ichthyosis follicularis, atrichia, and photophobia (IFAP, n = 1); ectrodactyly-ectodermal dysplasia-clefting (EEC, n = 1); and ichthyosis with confetti (IWC, n = 1). Foot problems not only varied in severity depending on the disease but also showed phenotypic heterogeneity among patients with the same condition. Foot deformities were most pronounced in patients with EEC (split foot) or IWC (contractures) and less severe in those with ARCI (clawed toes), IFAP (hollow feet), or PPK (no bone abnormalities in the feet).

**Conclusion:**

Because a range of distinct genodermatoses involve foot abnormalities, early rehabilitation and other corrective measures should be provided to patients with foot involvement to improve gait and prevent/delay irreversible complications.

**Supplementary Information:**

The online version contains supplementary material available at 10.1186/s13023-022-02207-x.

## Background

Many dermatological diseases cause substantial foot deformities that often require physical therapy or orthopaedic care [1]. Foot deformities are observed in many genodermatoses. In pachyonychia congenita, blisters and calluses often develop on the feet [2]. Palmoplantar keratodermas (PPKs), a heterogeneous group of cornification diseases, are characterised by a thickened epidermis on the palms and soles [3]. Some syndromic generalised diseases of cornification cause symptoms such as abnormal second and third toes (Refsum disease), pes cavus (ichthyosis follicularis, atrichia, and photophobia [IFAP]), or limb shortening (Conradi-Hünermann-Happle syndrome) [4, 5]. However, little data are available on foot deformities in rare genodermatoses; as such, foot deformities and their consequences in patients with genodermatoses are often neglected in clinical practice, reducing patients’ quality of life [6].

In this study, we assessed the presence of foot disorders in 16 patients with genodermatoses who attended our clinic.

## Patients and methods

Between April 2019 and December 2020, we enrolled patients with genetically confirmed genodermatoses, who were then evaluated for foot abnormalities in the Dermatology Clinic and Department of Paediatric Orthopaedics and Rehabilitation at the Medical University of Lublin. All patients gave informed consent to participate. We analysed each patient’s clinical history, with a particular focus on the type of foot disorders and their management.

In each patient, molecular analysis was performed to confirm the diagnosis. In two patients (P14 and P15), biopsies and molecular analyses were done as described previously [7, 8]. In the remaining patients, next-generation sequencing was carried out with a dedicated panel (NimbleDesign, Roche) of genes that cause Mendelian disorders of cornification (MeDOC; described in Additional file 1). The American College of Medical Genetics classification was used for variant interpretation [9]. The identified variants were confirmed with Sanger sequencing. Table 1 shows the variants found in the patients studied.

**Table 1:** Genetic analysis and skin lesions in patients with rare genodermatoses evaluated for foot abnormalities

Patient no		Phenotype	Skin lesions	Palmoplantar features	Gene (inheritance)	Genotype	Comment
1	Figure 1	ARCI-SICB	Ichthyosis vulgaris-like desquamation, erythema on cheeks	Hyperkeratosis and exaggerate decreases; palmar hyperlinearity	*ALOX12B (AR)*	NM_001139:c.[1156C > T];[1790C > A] NP_001130.1:p.[(Arg386Cys)];[(Ala597Glu)]	Known variants
2	Figure 1	ARCI-SICB	Ichthyosis vulgaris-like desquamation	Marked palmoplantar creases and desquamation	*ALOX12B (AR)*	NM_001139.2: c.1207C > T(;)1790C > A NP_001130.1:p.(His403Tyr)(;)(Ala597Glu)*	Known variants
3	Figure 1	LI/CIE Overlap-PI	CIE at birth. Then mild ichthyosis: whitish scales on trunk and proximal limbs and hyperkeratosis mainly on distal limbs. Lichenification around ankles and wrists	Mild hyperkeratosis on palms and soles with exaggerated creases	*ALOX12B (AR)*	NM_001139.2: c.[2036G > A];[2060A > G] NP_001130.1: p.[(Arg679His)];[(Tyr687Cys)]	HGMD/ClinVar-no data MAF: 0.00041%. ACMG: likely pathogenic
4	Figure 1	ARCI similar	Dry and flaking skin of the body, with pale-pink and flesh-coloured scales; linear skin fissures on the lower legs	Mild circular hyperkeratotic lesions on metatarsals slight keratosis of the heels with delicate desquamation	*ALOX12B (AR)*	NM_001139.2:c.[1330G > A];[ =] NP_001130.1: p.[(Val444Ile)];[ =]	HGMD/ClinVar-no data MAF: 0.007% ACMG: VUS. A second variant was not found
5	Figure 2	ARCI-LI	Born as CB. Later generalized, mild ichthyosis with whitish scales. Facial erythema	Mild hyperkeratosis on palms and soles with exaggerated creases	*ALOX12B (AR)*	NM_001139.2: c.[1562A > G];[2094C > A] NP_001130.1: p.[ (Tyr521Cys)];[ (Ser698Arg)]	p.Tyr521Cys: pathogenic; p.Ser698Arg: HGMD/ClinVar-no data; ACMG: likely pathogenic
6	Figure 2	ARCI-LI	Born as CB. Severe LI with dark-brown body scales and hyperkeratosis on extremities	Severe palmoplantar hyperkeratosis	*TGM1 (AR)*	NM_000359.2: c.788G > A(;)1135G > C NP_000350.1: p.(Trp263Ter)(;)(Val379Leu)*	Known variants
7	Figure 2	ARCI-LI	Born as CB. Moderate to severe lamellar-ichthyosis with generalized, dark-brown scales	Moderate palmoplantar hyperkeratosis	*TGM1 (AR)*	NM_000359.2:c.[337G > A];[=] NP_000350.1:p.[(Arg126His)];[=]	p.Arg126His. HGMD/ClinVar: likely pathogenic
8	Figure 3	Punctate PPK type I (Buschke-Fisher-Brauer)	None	Punctate areas of hyperkeratosis over bony prominences of the feet	*AAGAB (AD)*	NM_024666.4:c.[4G > A];[=] NP_078942.3:p.[(Ala2Thr)];[=]	HGMD/ClinVar-no data MAF: 0.002% ACMG: VUS
9	Figure 3	Striate PPK type I	None	Linear hyperkeratosis on palms and palmar aspects of the fingers, focal hyperkeratosis at pressure points on soles	*DSG1 (AD)*	NM_001942.3: c.[518-2A > G];[=] NP_001933.2: p.[?];[=]	HGMD/ClinVar-no data; ACMG: likely pathogenic
10	Figure 3	Diffuse PPK-Unna-Thost	None	Diffuse yellowish (non-transgredient) keratoderma with well-defined volar border	*KRT9 (AD)*	NM_000226.3: c.[488G > A];[=] NP_000217.2: p.[(Arg163Gln)];[=]	Known variant
11	Figure 3	Diffuse PPK-Unna-Thost			*KRT9 (AD)*	NM_000226.3: c.[488G > A];[=] NP_000217.2: p.[(Arg163Gln)];[=]	Known variant
12	Figure 3	Diffuse PPK-Greither disease	Patchy hyperkeratosis on elbows, knees, inguinal folds, and axillae	Moderate to severe, diffuse thick yellow/brownish hyperkeratosis on soles extending over Achilles’ tendons and extensor aspects of the feet and hands	*KRT1 (AD)*	NM_006121.4:c.[1535delT];[=] NP_006112.3: p.[(Ile512ThrfsTer102)];[=]	HGMD/ClinVar-no data; ACMG: likely pathogenic
13	Figure 3	Diffuse PPK-Greither disease	Fissures, hyperhidrosis. Patchy hyperkeratosis on elbows, knees, inguinal folds, and axillae		*KRT1 (AD)*	NM_006121.4:c.[1535delT];[=] NP_006112.3: p.[(Ile512ThrfsTer102)];[=]	HGMD/ClinVar-no data; ACMG: likely pathogenic
14	Figure 4	IFAP	Generalized follicular papules, sparse eyebrows and eyelashes, psoriasiform plaques in the intergluteal fold	Abnormal dermatoglyphics on plantar surfaces of feet	*MBTPS2 (XLR)*	NM_015884.4: c.[1001G > A];[0] NP_056968.1 p.[(Cys334Tyr)];[0]	Published previously
15	Figure 4	EEC	Sparse and brittle hair; dystrophic nails; psoriasiform lesions on the elbows; hypopigmented patches on trunk and extremities	Abnormal dermatoglyphic on plantar surfaces	*TP63 (AD)*	NM_003722.4: c.[941G > A];[=] NP_003713.3: p.[(Gly314Glu)];[=]	Published previously
16	Figure 4	IWC	Born as CB. Generalized ichthyosiform erythroderma with whitish spots of apparently normal skin; hypoplasia of mammillae, ear malformations, ectropion, sparse eyebrows and eyelashes, pruritus, and joint contractures	Moderate PPK	*KRT10 (AD)*	NM_000421:c.[1554dupC];[=] p.[(Ser519GlnfsTer62)];[=]	HGMD/ClinVar-no data; ACMG: likely pathogenic

“0”—the absence of a second X-chromosome (in males); = —no variant in the coding sequence of a second allele; “?”—effect on protein level is not known; all names and genotypes are according to HGVS nomenclature (* the separator (;) means that two variants in a gene were found, but parental analysis was not performed, hence it was not molecularly confirmed that they are on the different alleles); ACMG: Standards and guidelines for the interpretation of sequence variants: a joint consensus recommendation of the American College of Medical Genetics and Genomics and the Association for Molecular Pathology (Richards S, et al. Genet Med. 2015; 17:405–424). AR—autosomal recessive; AD—autosomal dominant; XLR—X-linked recessive; ARCI—Autosomal Recessive Congenital Ichthyosis; ARCI-SICB—Autosomal Recessive Congenital Ichthyosis: self-healing collodion baby; ARCI-LI/CIE-PI—Overlap Autosomal Recessive Congenital Ichthyosis: lamellar ichthyosis/congenital ichthyosiform erythroderma-pleomorphic ichthyosis; ARCI-LI/PI—Autosomal Recessive Congenital Ichthyosis: lamellar ichthyosis/pleomorphic ichthyosis; ARCI-LI—Autosomal Recessive Congenital Ichthyosis: lamellar ichthyosis; CB—Collodion baby; CIE—Congenital ichthyosiform erythroderma; IFAP—Ichthyosis follicularis, alopecia, and photophobia syndrome; EEC—Ectrodactyly, ectodermal dysplasia, and cleft lip/palate syndrome; IWC—Ichthyosis with confetti; PPK—Palmoplantar Keratoderma; VUS—Variant of unknown significance; MAF—minor allele frequency

## Results

### Autosomal recessive congenital ichthyosis

We included seven patients with autosomal recessive congenital ichthyosis (ARCI) with various foot deformities (Figs. 1, 2; P1–7).

**Fig. 1:**
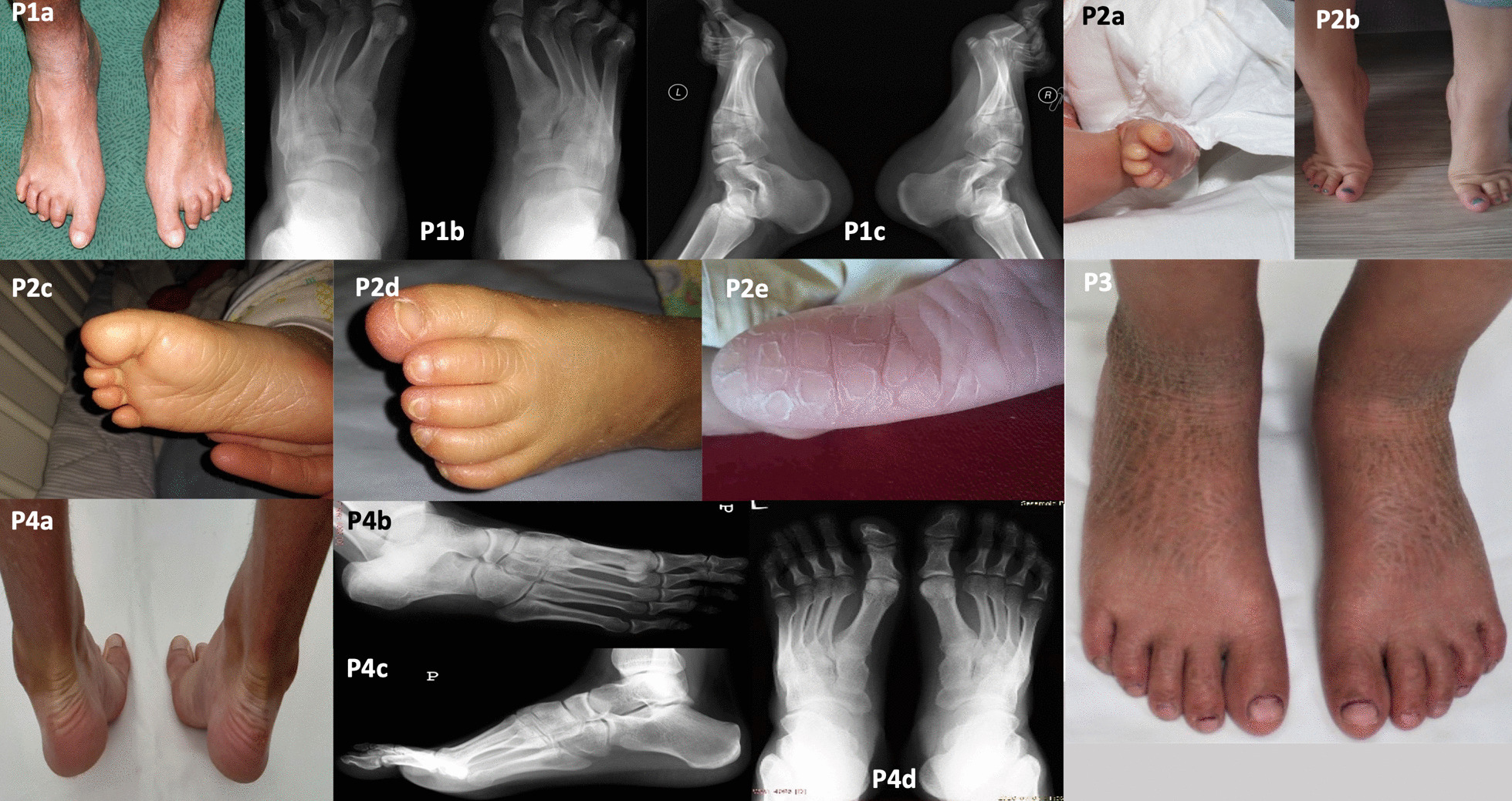
Patients 1–4 with autosomal recessive congenital ichthyosis. Pes cavus, pes varus, toe contractures, hollow feet, widened forefeet, claw toes II–V, and varus heels rotated inwards are visible on a photograph (P1a) and radiographs (P1b, P1c). Toe contractures at birth (P2a). Morphologically normal feet, but early signs of claw and varus toes (toes III–V overlapping) were seen when standing on tiptoes (P2b–c). Skin cracks on the soles (P2d–e) with plaques and fissures (P2f). Normal feet (P3). Normal feet clinically (P4a) and radiologically (P4b–c)

**Fig. 2:**
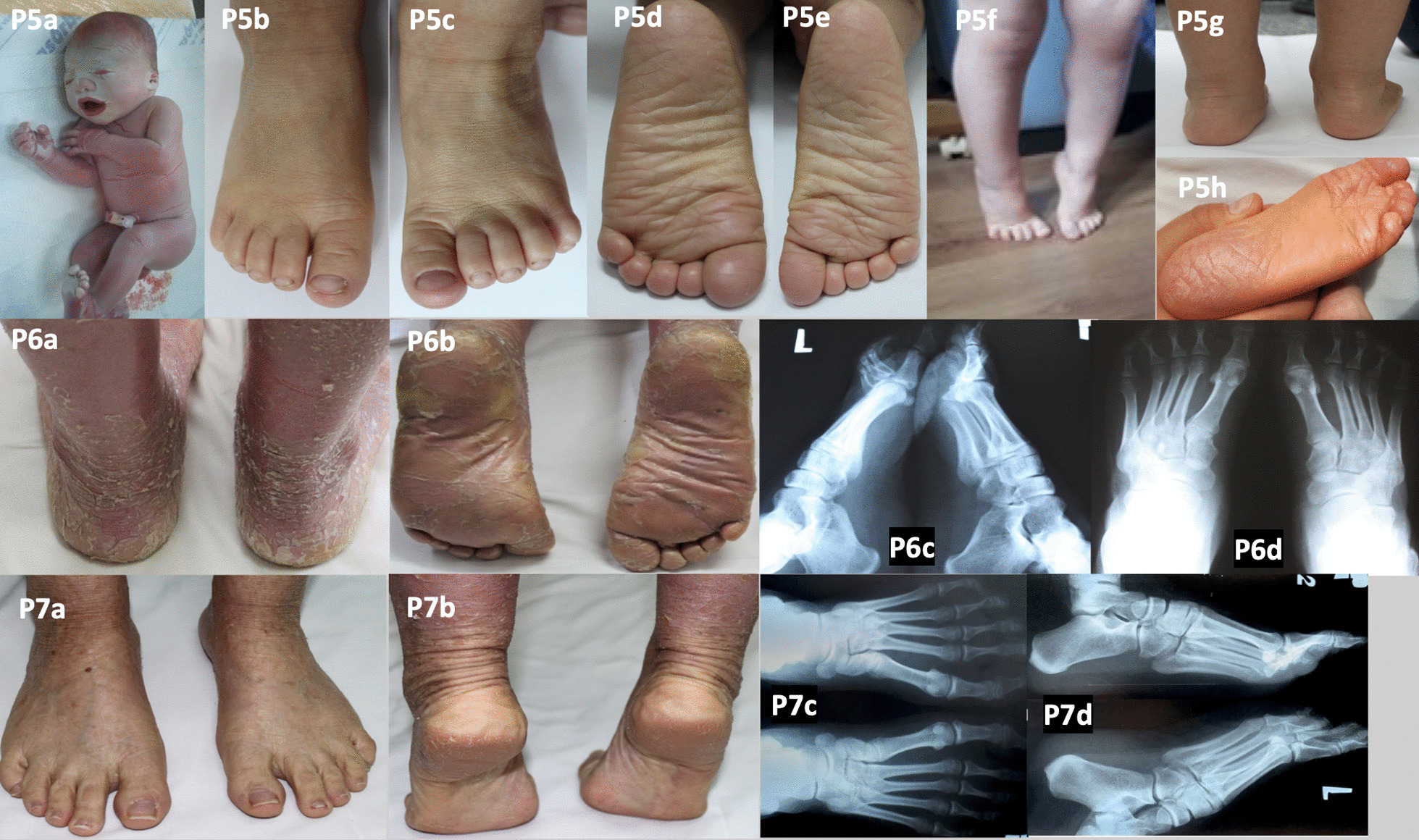
Patients 5–7 with autosomal recessive congenital ichthyosis. Collodion baby (P5a). Skin lesions on the dorsal aspect of the feet (P5b–c). Fissuring on the soles (P5d–e, h). Normal feet (P5f) and standing on tiptoes (P5g). Circular hyperkeratotic lesions on the ball of the foot, slight keratosis of the heels (P6a–b), clawed toes II–IV, and overlapping toes IV–V (P6c–d). Hyperkeratotic skin lesions on the soles (P7a–b). Normal X-rays of the feet (P7c–d)

First patient had pes cavus, pes varus, toe contractures, hollow feet, claw toes II–V (normal great toe), and varus heels (Fig. 1; P1a–c). These abnormalities were confirmed on X-ray (Fig. 1; P1b–c). Moreover, the patient had high-arched, neuropathic feet and widened forefeet. Body posture and gait were normal but walking in the sand or sudden movements of the toes often caused skin fissures. He often had callosities on the outermost toes, with abnormal nails. Due to the position of the feet and toes, the patient had problems finding footwear. We recommended orthopaedic insoles and physical therapy.

Patient 2 (niece of the previous patient) had morphologically normal feet, but there were early signs of claw and varus toes (toes III–V overlapping) and hallux valgus when standing on tiptoes (Fig. 1, P2a–e). We recommended wide shoes with a soft insole and a consultation with an orthopaedic surgeon.

We did not observe any radiological foot abnormalities in the next three patients with ARCI (P3–5; Table 1, Figs. 1, 2).

In patient 6 we observed foot deformities: claw toes II–IV (metatarsophalangeal joint hyperextension and increased flexion in the interphalangeal joints) and overlapping toes IV–V (Fig. 2; P6a–b). Her gait was also impaired because of deep skin cracks on the heels. She complained of knee pain (more pronounced on the right), mainly when walking, sitting for a long time, crouching, or standing up. An X-ray of the knee showed a narrowed joint space with increased sclerosis of the joint surfaces. The knee pain decreased after supplementation with chondroitin sulphate and an adequate diet.

Patient 7 had scaling skin lesions, often complicated by poorly healing wounds, on the dorsal and plantar sides of the feet (Fig. 2; P7a–d). An X-ray of the knee joints showed slightly narrowed joint spaces on both sides, sharpened intercondyloid eminence of the tibia, and lateralisation of both patellae.

### Palmoplantar keratodermas

**Fig. 3:**
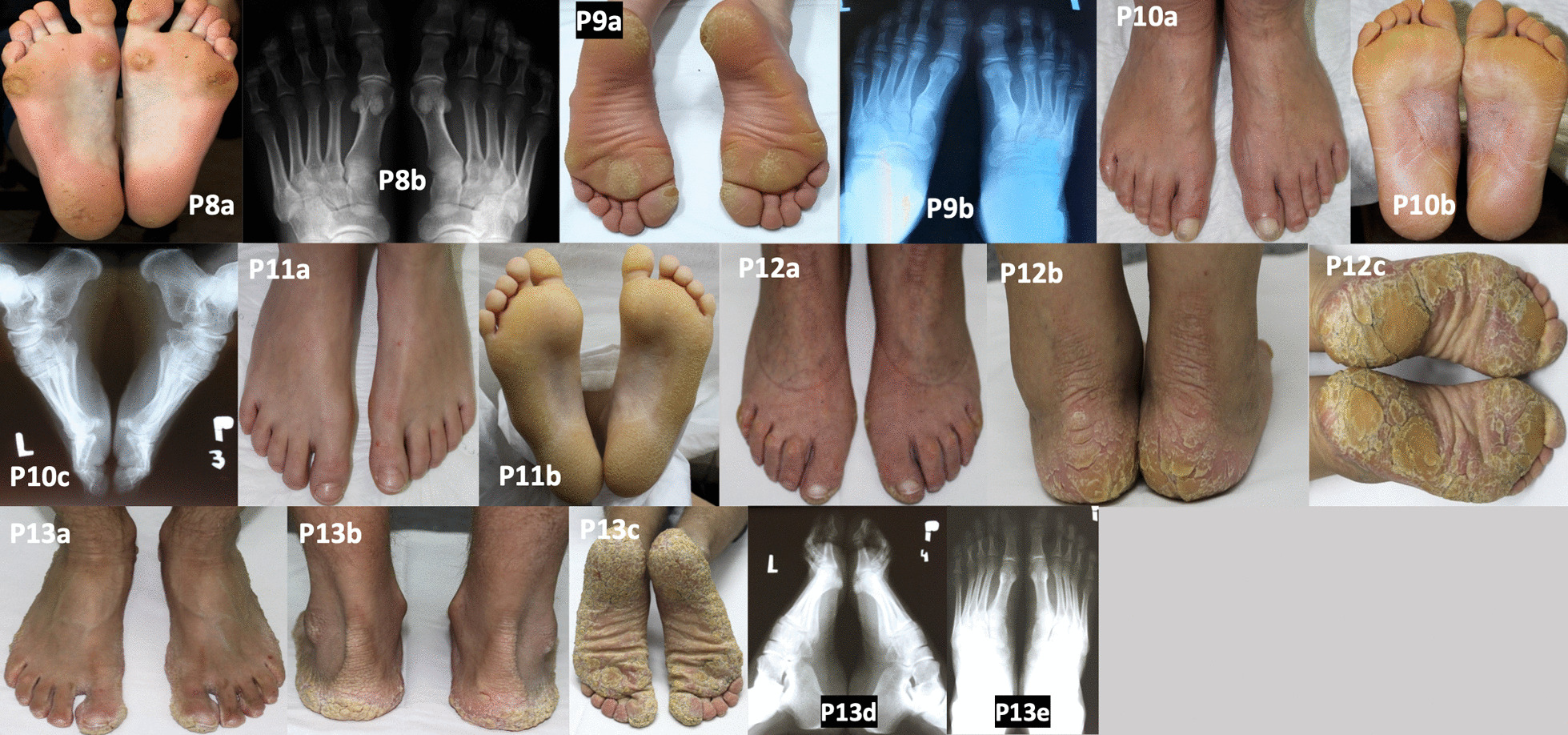
Patients with palmoplantar keratodermas. Areas of hyperkeratosis on the heels and foot arches and skin cracks on the right heel (P8a). Normal feet on a radiograph (P8b). Round areas of hyperkeratosis on the soles (P9a). Normal feet on a radiograph (P9b). Well-defined epidermal hyperkeratosis, numerous cracks in the epidermis arranged lenticularly, without fissures (P10a–b). Heel spur on a radiograph (P10c). Healthy skin on the dorsal feet (P11a). Well-defined enhanced epidermal hyperkeratosis and numerous cracks arranged reticularly, without fissures (P11b). Mild hallux valgity, claw toes II–V, with hyperkeratosis over interphalangeal joints II–V (P12a). Accentuation of the sulci over the Achilles tendon (P12b–c). Thick scaling on the skin of heels, soles, and metatarsal area, passing onto the forefoot in an arched manner, but not affecting the medial longitudinal arch of the foot (P12c). Healthy skin on the dorsal feet (P13a). Excessive epidermal furrowing and enhanced keratosis on the skin of the heels and over the Achilles tendon (P13b), with increased lesion severity on the skin of the heels, lateral surfaces of the foot, the entire forefoot, and the skin of the big toes (P13c). Normal foot radiographs (P13d–e)

Patient 8 was presented with punctate PPK type I (Buschke-Fischer-Brauer, autosomal dominant) [10]. She had bilateral pes planus and hallux valgus. She required metatarsal insoles to support the metatarsal bones. The longitudinal arch of the foot was normal, but varus tarsus was visible. An X-ray showed no radiological foot changes (Fig. 3; P8a–b). She had normal posture, but her gait was characterised by a tendency to protect the damaged parts of the foot, which caused a shift in the centre of balance. When walking, the patient off-loaded the 5th metatarsal head area in the right foot, which resulted in an overload in the medial foot aspect. She also felt foot pain when standing for a long time, walking down the stairs (but not climbing up the stairs), or walking on a flat surface for a long time. When working in the garden, she preferred to be on her knees to avoid foot pain. Her mother died of bladder cancer. We are currently conducting cancer screening in this patient.

Patient 9 with striate PPK type I had foot pain when standing, using the stairs, or walking on a flat surface (Fig. 3; P9a–b). The patient had abnormal posture, with pelvic anteversion, and shortened triceps surae muscles, with limited dorsiflexion. Due to the COVID-19 pandemic, the boy’s mother did not agree to his hospitalisation, but stretching exercises were initiated, which improved his orthopaedic condition.

We found no bone abnormalities in the feet except for a heel spur (visible on X-ray), which was unrelated to the main disease in other patient with autosomal dominant diffuse PPK (Thost-Unna or Vorner type) (Fig. 3; P10a–c). However, she experienced problems when soaking her feet in sea water or in a swimming pool, which caused dry skin and softening of the epidermis. When her feet were soaked, the patient felt pain when walking (e.g. barefoot on a sandy beach or when wearing footwear with plastic elements). The patient complained of hyperesthesia of the feet. Her teenage daughter (P11), who was also diagnosed with PPK, did not have any foot abnormalities (Fig. 3; P11a–b).

Patient 12 with the most clinically severe diffuse PPK phenotype (transgrediens et progrediens PPK, Greither disease) had toe contractures and severe scaling and dry skin lesions on the feet (Fig. 3; P12a–c). She presented with radiological foot abnormalities. Her son (P13), who was also diagnosed with Greither disease, did not have any radiological foot abnormalities, although his phenotype was severe (Fig. 3; P13a–e). Both patients experienced an exacerbation of local changes after stress.

### Other genodermatoses

**Fig. 4:**
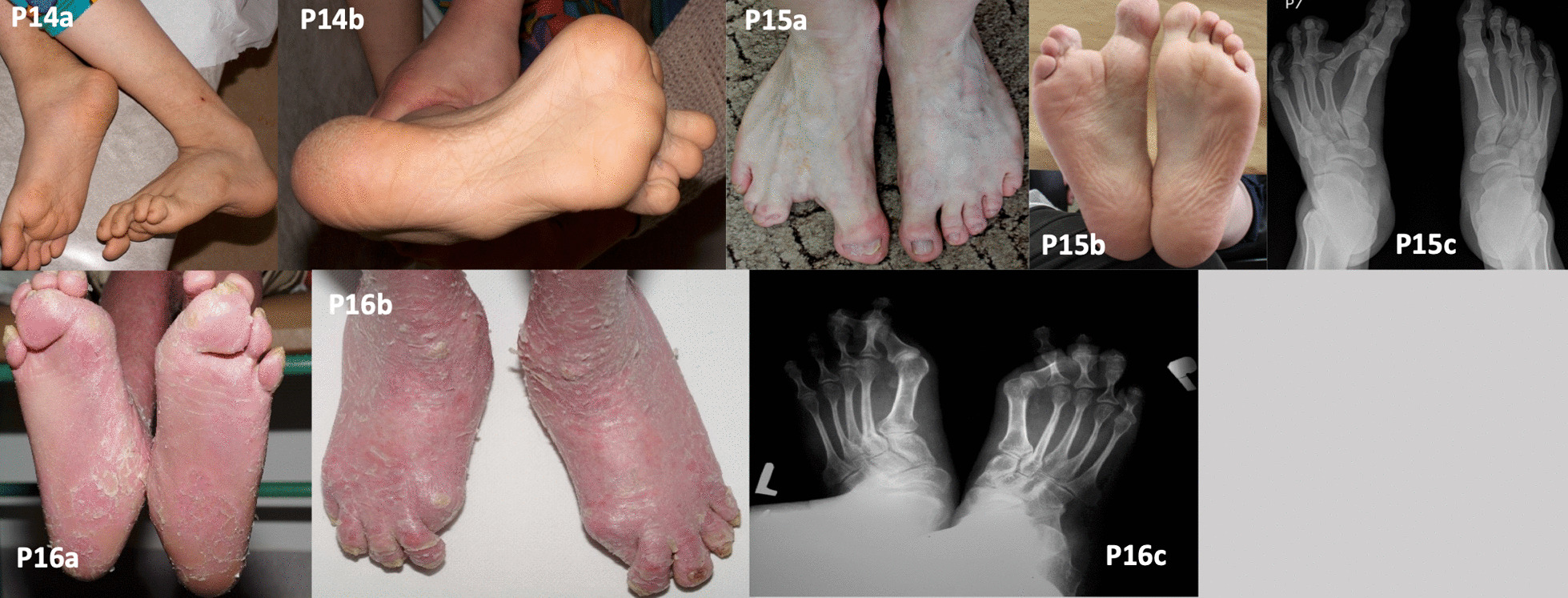
Other genodermatoses. Clubfoot in the left foot (P14a). Claw toes (P14b). Split foot deformity in a patient with ectrodactyly-ectodermal dysplasia-clefting syndrome (P15a–b). A radiograph (P15c) shows the additional residual middle phalanx of digit III in the right foot, deformed distal phalanges of digits IV and V, syndactyly digits III–IV and IV–V, a shortened middle phalanx of digit V in both feet, and the abnormal position of the distal interphalangeal joints. Hallux valgus, angling inward under toes II–IV of the right foot, and under toes II and III of the left foot, in a patient with ichthyosis with confetti (P16a–b). The same abnormalities are visible on a radiograph (P16c). Figure P16c has been previously published by Wawrzycki et al. [8] it is Open Access article distributed under the terms of the Creative Commons Attribution-NonCommercial-ShareAlike 4.0 International (CC BY-NC-SA 4.0) License

Patient 14 patient was diagnosed with X-linked IFAP. Clinical and histopathological findings in this patient have been published previously [8]. He presented with multiple concomitant skeletal malformations including high-arched (cavus) feet (Fig. 4; P14a–b). Additionally, we noticed deformities of both femoral bones (epiphyseal-metaphyseal), patella lateralisation, and thinning of the fibulas. The patient was unable to lift his feet properly and walked with support because of problems with balance, disturbed muscle tone, and weakness in both lower limbs. The patient needed to use soft silicone insoles for support of the entire foot and toes, with silicone toe fillings, which were custom-made by foot casting.

Patient was diagnosed with autosomal dominant EEC confirmed by a novel p.Gly314Glu variant in the *TP63* gene, as published previously [7]. The patient presented the hallmark signs of this syndrome—i.e. split foot/hand deformity and syndactyly of digits III–IV and IV–V (Fig. 4; P15a–c). The distal phalanges of digits IV and V were deformed. In both feet, the middle phalanx of digit V was shortened, and the surfaces of the distal interphalangeal joints were positioned abnormally. An X-ray showed an additional residual middle phalanx of digit III in the right foot, which was horizontally arranged and connected to the metatarsophalangeal joint with an additional joint surface. The patient did not consent to surgical treatment due to the risk of foot pain and gait impairment associated with the procedure. To our knowledge, this was the first reported case of a patient with EEC and co-existing cutaneous lesions. Other clinical and laboratory details of this patient have been published previously [7].

Patient 16 with ichthyosis with confetti (IWC; P16) had knee valgity that exceeded 15 degrees, affecting the position of the feet (Fig. 4; P16a–c). His gait was broad-based, with characteristic side movements of the pelvis (pelvic anteversion) and foot dragging due to pain in the forefoot, particularly in the area of the metatarsophalangeal joint of the big toe. His gait was impaired by foot and leg deformities (particularly in the forefoot), joint contractures, and poor coordination. Treatment included orthopaedic shoes, which improved the patient’s gait substantially and reduced contractures.

## Discussion

Herein, we present a spectrum of skin lesions and foot deformities in 16 patients with rare genodermatoses. Because problems related to foot abnormalities are often overlooked in dermatology practice, patients with genodermatoses often suffer from foot pain and functional gait impairment. This case series may help to identify specific problems associated with foot abnormalities in patients with rare genodermatoses to prevent/delay irreversible complications and improve patients’ quality of life. Most of our patients suffered from various degrees of foot hyperkeratosis that belong to generalised or localised MeDOC, ichthyoses, or PPKs.

Nearly half of the patients were diagnosed with ARCI, a genetically heterogeneous group of disorders that present with overlapping clinical features [11, 12]. We identified a full genotype in five patients with ARCI, whereas two patients (P4 and P7) had variants detected in only one allele of either the *ALOX12B* or *TGM1* genes. Previous reports indicate that patients with *TGM1* mutations are generally more severely affected with more frequent collodion membrane at birth, ectropion, plate-like scales, and PPK [11–13] than those with *ALOX12B* mutations. Thus, our results seem to be in concordance with those of previous studies, suggesting that the more severe phenotype of individuals with mutations in *TGM1* also extends to palmoplantar involvement.

Patients with ARCI are often born covered with a parchment-like membrane, a phenotype known as collodion baby [14]. Most patients with ARCI in our series were born as collodion babies (except for P4). The tautness of the skin results in a striking clinical appearance with ectropion, eclabium, and sometimes pseudocontractures or oedematous extremities (Fig. 2; P5a) [15]. Our observations indicate that, regardless of genotype, each patient with ARCI deserves an individualised assessment of foot abnormalities to select the best treatment. Management includes topical treatment of skin lesions (e.g. moisturisers, keratolytics, or retinoids), orthopaedic shoes for foot deformities, physical therapy for contractures, and surgery when conservative treatment fails.

There were six patients with PPK in our series. PPKs are heterogeneous cornification diseases characterised by thickened epidermis on the palms and soles. Our patients with localised MeDOC had three major clinico-morphological patterns of PPK: Thost-Unna/Vorner type (MIM no. 144200/600962), Greither disease (MIM no. 144200), focal/striate PPK type I (MIM no. 148700), and punctate PPK type I (Buschke-Fischer-Brauer; MIM no. 148600/614936) [12]. These patients presented with the typical features of PPK. The two patients with Thost-Unna/Vorner type had diffuse yellowish compact hyperkeratosis [3] accentuated on pressure-bearing points, implying that this type of PPK might also be aggravated by mechanical trauma (Fig. 3; P10a–c, P11a–b). The two patients with Greither disease had the most severe phenotype, with thick, brownish hyperkeratosis, recurrent fissures, hyperhidrosis, and features of progredience and transgredience (Fig. 3 P12 a–d, P13 a–e) [3]. The boy with striate PPK type I had focal/nummular plantar hyperkeratosis, mainly at sites of mechanical pressure, as previously described (Fig. 3; P9) [16]. The woman with punctate PPK type I displayed hyperkeratotic papules coalescing into larger callus-like lesions on weight-bearing areas of plantar skin, which were associated with pain (Fig. 3; P8). Strikingly, in large case series of punctate PPK type I, plantar pain was reported in 12 of 16 investigated patients [17]. Our patients with PPKs did not have major foot abnormalities (P13 and P14). One patient had abnormal posture, due to pelvic anteversion, and shortened muscles of the lower extremity. Another patient tended to off-load the areas of the foot with skin lesions when walking, which resulted in an abnormal gait. However, PPK did not impair the gait functionally. Our patients with PPK were successfully treated with topical moisturisers and keratolytics. In patients with Greither disease, foot changes were visible on X-rays only, and included hallux valgus and claw toes II–V. There is no specific or curative therapy available for PPKs. Most treatments are symptomatic and offer only temporary relief. Local symptomatic treatment of PPKs involves three components: keratolytics, moisturisers, and mechanical debridement. Nevertheless, severe PPKs localised on the whole plantar surface may require surgery (keratoma excision) [18].

The patient with keratinopathic ichthyosis (IWC type I) had moderate sole hyperkeratosis with erythema and desquamation as the predominant manifestations (Fig. 4; P16). Similar to other described cases, PPK of varying severity is associated with most cases of IWC type I (*KRT10*), whereas disproportionately severe hyperkeratosis has been reported in IWC type II (due to *KRT1*) [19]. Patients with IWC are born with generalised ichthyosiform erythroderma or as collodion babies, and later develop small confetti-like spots due to revertant mosaicism [19]. Our patient displayed the typical features of the disease: collodion baby phenotype, palmoplantar hyperkeratosis, and confetti-like spots. Additionally, joint contractures, knee valgity, and pelvic anteversion impaired his gait substantially. As in other genodermatoses, patients with IWC and foot deformities should be under the care of a multidisciplinary team of neurologists and orthopaedic or plastic surgeons. Other investigators reported increased peripheral reflexes and psychomotor retardation in patients with IWC [20]. Our patient needed only to use soft silicone insoles for foot and toe support, which were custom-made by foot casting.

IFAP is a rare X-linked genetic disorder caused by pathogenic *MBTPS2* variants. Although the number of reported IFAP cases is about 40, we presume that the actual number of cases is greater, most of which present with additional features such as atopic eczema [21]. The one patient with IFAP in our current series had severe skeletal malformation including high-arched (cavus) feet, which impaired walking (Fig. 4; P14).

Our patient with EEC type 3 (P15) had a variant in the *TP63* gene. EEC is a rare autosomal dominant syndrome belonging to the group of ectodermal dysplasias [22]. The disease manifests with numerous symptoms of varying severity. The clinical presentation in our patient included the typical symptoms of plantar hyperkeratosis, dystrophic nails, and split foot. Although the patient moisturised his feet and soaked them daily to minimise the risk of trauma, he developed bacterial and fungal infections of the feet. This patient needed to wear customised shoes. Patients with EEC and foot deformities may undergo surgery to remove problematic bone fragments and to straighten the longitudinal axis of the toe [23]. However, the patient declined surgical treatment.

The predominant problems of the patient with IFAP were neurological, whereas the predominant problems of the patient with EEC were orthopaedic. Otherwise, both patients had exaggerated and disturbed dermatoglyphic sole patterns, which, to our knowledge, has previously been described in only single case reports [7, 24]. For example, the palms and soles are generally unaffected in IFAP [25], with one reported case of associated plantar keratoderma [26]. In EEC, the skin is usually fine, dry, and of atopic appearance [27]. Interestingly, ankyloblepharon-ectodermal defects-cleft lip/palate (AEC, OMIM 106,260), another EEC-like syndrome caused by mutations in the gene encoding the p63 transcription factor, is well known to cause palmar and plantar changes with effaced dermatoglyphics [28].

**Fig. 5:**
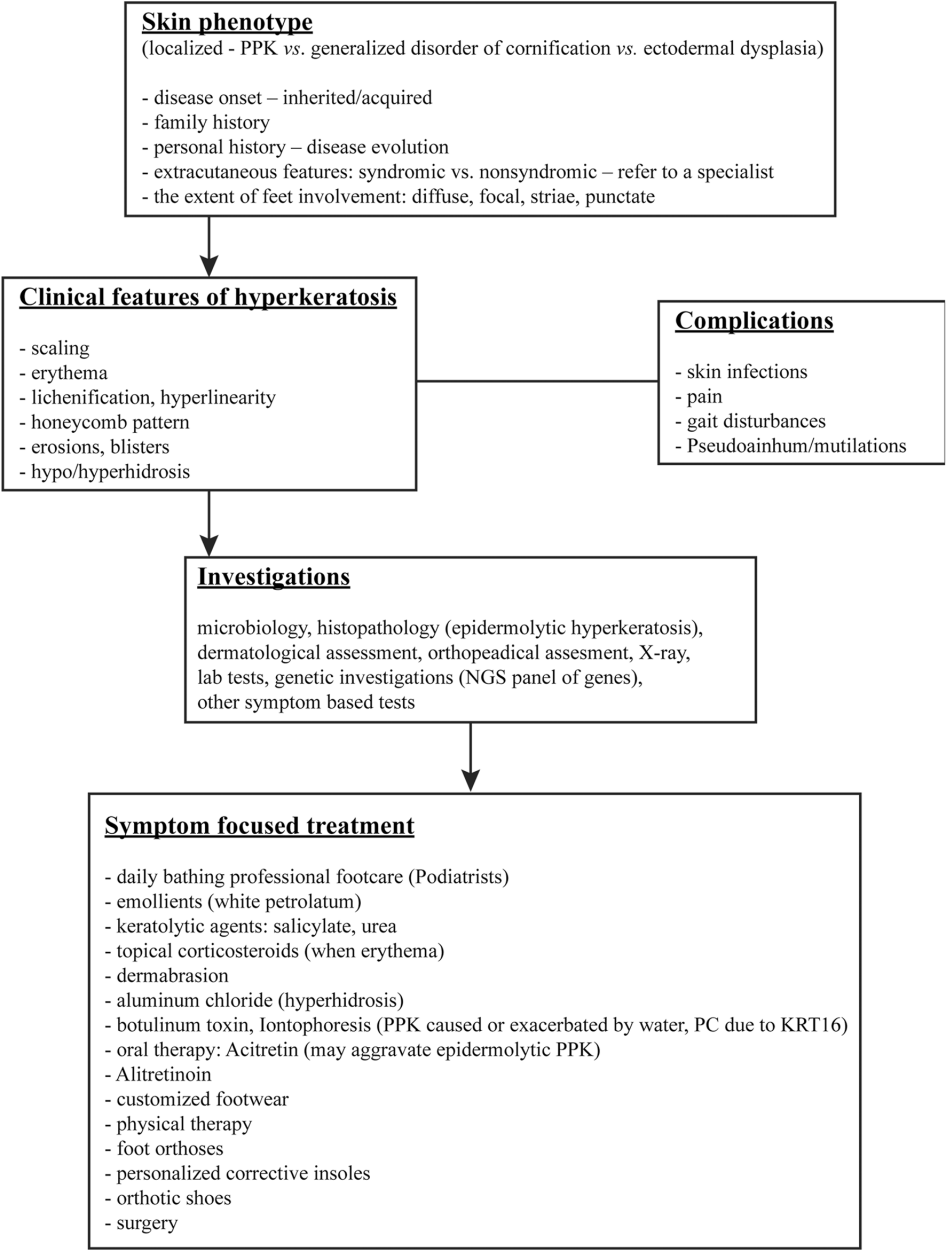
Management algorithm for patients with genodermatoses. NGS—next-generation sequencing; PC—pachyonychia congenita; PPK—palmoplantar keratodermas

In this work, we concentrated on rarely discussed foot deformations among patients with various genodermatoses. In most forms of the presented MeDOCs, foot function is largely maintained. Nevertheless, plantar hyperkeratosis with fissures or focal keratoderma can be particularly painful, resulting in difficulties in walking, hindering the affected individuals’ everyday lives [3]. Therefore, we strongly recommend asking for an orthopaedic consult as a standard procedure in these patients. In Fig. 5, we propose a management algorithm for patients with genodermatoses.

Many foot deformities have a substantial effect on patients’ lives. Treatment should be selected individually for each patient, and may include management of skin lesions, physical therapy, foot orthoses, personalised corrective insoles, orthotic shoes, or surgery. Footwear should be properly selected and stretched. Otherwise, patients may develop painful foot syndrome, which causes further gait deterioration. Of note, surgical treatment may also exacerbate secondary deformities and degenerative changes, especially in patients with skin diseases. The mechanism underlying pain in many types of PPK remains poorly understood. It is still reported inconsistently and most probably treated inadequately [29].

Topical therapy is currently the mainstream of both ichthyoses and palmoplantar keratodermas treatment. This approach may relieve symptoms and eventually prevent sequelae such as contractures. The choice of the drug and frequency of its use depends on the type and severity of disease. The local symptomatic treatment of hyperkeratosis of the feet involves keratolytics, moisturizers, and mechanical debridement. Keratolytic preparations include various concentrations of salicylic acid, lactic acid, urea, and propylene glycol. Both topical and systemic retinoids are another pillar of management. Tretinoin, adapalene, and tazarotene are applied topically, while isotretinoin and acitretin are used systemically. In addition, topical steroids and calcipotriol have been used with varying results.

Local infections and hyperhidrosis should be treated when needed. There are few reports of plastic surgery interventions in patients with PPKs. More specific therapies in the form of nonsense suppression (readthrough), short interfering RNA (siRNA), and genome editing tools (CRISPR/Cas9) are currently under investigation. Recent studies have uncovered T helper type 17 skewing in ichthyotic skin, resembling psoriasis, opening new avenues for the more targeted therapies [30].

## Conclusions

Treatment of genodermatoses is challenging and requires an interdisciplinary approach. Even if symptoms are subtle, early rehabilitation and corrective shoes should be provided to improve gait and prevent/delay irreversible complications.

## Supplementary Information

**Additional file 1**: Next-generation sequencing performed in patients 1 and 4–16

## Data Availability

Data are available upon request.
