# Supplementary material for: Structural and functional foot disorders in patients with genodermatoses: a single-centre, retrospective chart review
Source: Orphanet J Rare Dis. 2022 Feb 16;17:53. doi: 10.1186/s13023-022-02207-x (PMC8848968; doi:10.1186/s13023-022-02207-x)
Supplement: Supplementary file 1 — Additional file 1: Next-generation sequencing performed in patients 1 and 4–16 [file 13023_2022_2207_MOESM1_ESM.docx]

Supplement 1

Next-generation sequencing performed in Patients 1-13,16.

NimbleDesign, Roche panel of Mendelian disorders of cornification-causative genes (coding exons of *AAGAB, ABCA12, ABHD5, ADAM10, ALDH3A2, ALOX12B,ALOXE3, AP1S1, AQP5, CDSN, CLDN1, CSTA, CTSC, CYP4F22, DSG1, DSP, EBP, ENPP1, ERCC2, ERCC3, FERMT1, FLG* [fragment covering amino acids 1-2200], *GJA1, GJB2, GJB3, GJB4, GTF2H5, HOXC13, JUP, KANK2, KRT1, KRT10, KRT16, KRT17, KRT2, KRT9, LIPN, LOR, MBTPS2, MPLKIP, NIPAL4, NSDHL, PEX7, PHYH, PKP1, PNPLA1, POFUT1, POGLUT1, POMP, SERPINB7, SLC27A4, SLURP1, SNAP29, SPINK5, ST14, STS, SUMF1, TGM1, TRPV3, VPS33B*). Briefly, libraries were prepared using the KAPA Library Preparation Kit (Roche) and sequenced on the MiSeq (Illumina) according to the manufacturer’s instructions. The results were analysed with the following bioinformatic tools: Real-Time Analysis Software (RTA), MiSeq Reporter (MSR), VariantStudio, and Annovar and annotated using the SNPdb (NCBI), ExAC, Ensembl, OMIM, GnomAD, ClinVar, and HGMD Professional.
